# Genome-wide prediction methods for detecting genetic effects of donor chromosome segments in introgression populations

**DOI:** 10.1186/1471-2164-15-782

**Published:** 2014-09-11

**Authors:** Karen Christin Falke, Gregory S Mahone, Eva Bauer, Grit Haseneyer, Thomas Miedaner, Frank Breuer, Matthias Frisch

**Affiliations:** Institute of Agronomy and Plant Breeding II, Justus Liebig University, 35392 Giessen, Germany; Plant Breeding, Technische Universität München, 85354 Freising, Germany; State Plant Breeding Institute, Universität Hohenheim, 70593 Stuttgart, Germany; Institute for Evolution and Biodiversity, Westfälische Wilhelms-Universität Münster, 48149 Münster, Germany; KWS Saat AG, Grimshelstr. 31, 37555 Einbeck, Germany

## Abstract

**Background:**

Introgression populations are used to make the genetic variation of unadapted germplasm or wild relatives of crops available for plant breeding. They consist of introgression lines that carry small chromosome segments from an exotic donor in the genetic background of an elite line. The goal of our study was to investigate the detection of favorable donor chromosome segments in introgression lines with statistical methods developed for genome-wide prediction.

**Results:**

Computer simulations showed that genome-wide prediction employing heteroscedastic marker variances had a greater power and a lower false positive rate compared with homoscedastic marker variances when the phenotypic difference between the donor and recipient lines was controlled by few genes. The simulations helped to interpret the analyses of glycosinolate and linolenic acid content in a rapeseed introgression population and plant height in a rye introgression population. These analyses support the superiority of genome-wide prediction approaches that use heteroscedastic marker variances.

**Conclusions:**

We conclude that genome-wide prediction methods in combination with permutation tests can be employed for analysis of introgression populations. They are particularly useful when introgression lines carry several donor segments or when the donor segments of different introgression lines are overlapping.

## Background

If the genetic variability for traits of agronomical interest is limited, plant breeders attempt to make available favorable alleles from exotic material in breeding programs. A main problem is that lines derived from crosses of elite and exotic parents lack adaptation and their agronomic performance is so poor that they cannot be directly used in the breeding process. So called introgression libraries or introgression populations [1] are a concept that tries to overcome the problem by establishing introgression lines, of which the genome originates in large part from an elite line and only small chromosome segments originate from an exotic donor. The goal of this concept is to generate lines that have the adaptation and agronomic performance of the elite parent, and are enhanced by small chromosome segments from the exotic donor, which provide favorable alleles for specific traits that should be improved.

Introgression populations have been developed first in tomato [2] and subsequently in other crops [3–6]. In most experiments [5–13] the Dunnett test [14] was used to detect whether an introgression line differs significantly from the recipient elite line. If a line, that is significantly better than the recipient with respect to a certain trait, contains only one single donor chromosome segment, then such an analysis is able to identify this segment as affecting the trait. However, the lines of an introgression populations typically carry more than one donor segment [5, 15]. For such introgression lines, the Dunnett test is not able to identify which of the donor segments affects the trait.

A linear model in which each donor segment has a fixed effect [16], can be used to analyse introgression popualtions with lines that carry more than one donor segment. It can be employed, if the number of donor segments in the introgression library does not surpass the number of introgression lines, i.e., if the design matrix of the linear model has full rank. For introgression populations, in which the number of donor segments exceeds the number of introgression lines, the donor segment effects are not estimable with a fixed linear model. Statistical analysis methods for such situations were not yet investigated.

The goal of our study was to investigate the usefulness of statistical methods developed in the context of genome-wide prediction for the analysis of introgression populations. In particular, our objectives were to (1) apply the BLUP [17] and RMLV [18] methods to simulated and experimental data, (2) investigate their power of detecting donor chromosome segments that have effects on the phenotype of an introgression line, as well as their false positive rate, and to (3) draw conclusions on their potential application for the analysis of introgression populations.

## Methods

### Estimating donor segment effects

The genetic effects of the donor segments on a phenotypic trait were estimated with the linear model **y**=**1***β*_0_+**Zu**+**e**. Here, **y** is the vector of the phenotypic values of *N* introgression lines, *β*_0_ a fixed intercept, **Z** the design matrix relating the donor segments to the introgression lines, **u** the vector of the donor segment effects, and **e** the vector of residuals.

**Figure 1:**
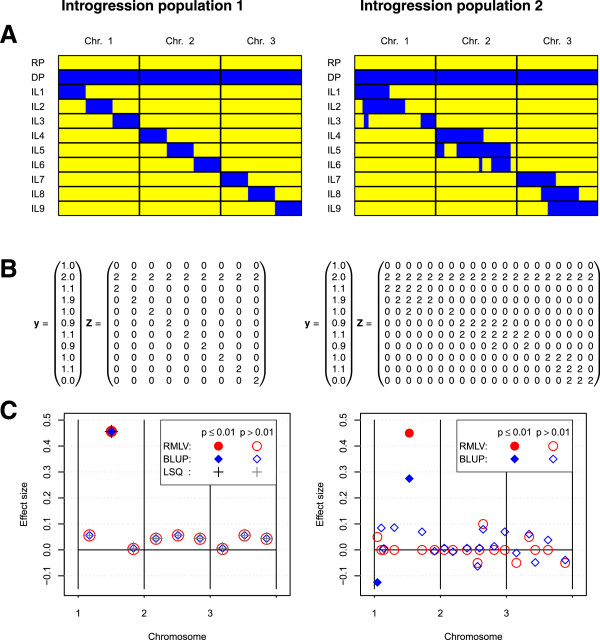
**Estimation of donor segment effects.** **A**: Graphical genotypes of two hypothetical introgression populations. **B**: The observation vector **y** contains phenotypic values and the design matrix **Z** illustrates the coding of the desing matrix for the two introgression populations. **C**: Estimated effect sizes and significances for effect estimation with an LSQ analysis (introgression population 1 only) and RMLV and BLUP analyses (both introgression populations).

To construct the the design matrix **Z**, markers for which the alleles were in complete linkage disequilibrium in the introgression population were combined to donor segments. The elements of **Z** are coded in the design matrix such that the number represents the donor segment zygosity, i.e., as 0, 1, 2. The structure of the design matrix **Z** is illustrated in Figure 1B for the two hypothetical introgression populations shown in Figure 1A.

For estimation of the donor segment effects, we used (a) least squares estimation (LSQ) assuming fixed donor segment effects, (b) best linear unbiased prediction (BLUP) assuming that the donor segment effects were random [17], or (c) the RMLV method suggested for genome-wide prediction [18]. For the LSQ analysis the intercept *β*_0_ was removed from the model. Calculations were carried out with the software SelectionTools (www.uni-giessen. de/population-genetics/downloads).

### Testing donor segment effects

For the LSQ analysis, the significance of the donor segment effects was tested with F-tests for linear contrasts. For the BLUP and RMLV analyses, we adopted a permutation test similar to that suggested by [19] for QTL mapping. For carrying out the permutation test for the effect *u*_*i*_ of the *i*th donor segment, entries of the *i*th column of **Z** were randomly permuted and *u*_*i*_ was estimated for the random permutations. The distribution of the *u*_*i*_ from *r* random permutations was used to approximate the distribution under the null-hypothesis that ‘the segment has no effect on the phenotype’. Comparison of the effect estimate obtained for the actually observed phenotypic data with the approximated distribution of effects under the null hypothesis was used to assign *p*-values to the donor effect estimates. The *p*-values from testing linear contrasts and from the permutation test were adjusted with a modified Bonferroni procedure [20].

### Sample data sets

For investigating effect estimation in introgression populations with genome-wide prediction methods, we considered two hypothetical introgression populations of different genetic structure. The genome considered for the simulations consisted of three chromosomes of length 120 cM. The introgression population 1 was an ideal introgression population consisting of 9 lines, each carrying a donor segment of length 40 cM. The donor segments were not overlapping. In introgression population 2 the donor segments had varying length, were overlapping, and several donor chromosome regions were present in more than one line. The graphical genotypes of both introgression populations are shown in Figure 1A.

For a first analysis we considered one major gene located in the center of chromosome 1 with an additive effect of size 0.5. An observation vector **y** that results from this genetic effect and a random error is shown in Figure 1B.

### Simulations for comparing power and false positive rate

We carried out computer simulations with the introgression populations 1 and 2 to determine the power and false positive rate of the LSQ, BLUP, and RMLV analyses. We simulated a quantitative trait, controlled by 2, 4, or 6 loci with additive gene action. The donor had a performance that was 100 units better than the recipient, hence, the effect of a favorable allele was 25, 12.5, and , respectively. The genes were assigned to random positions in the genome. Heritabilities between 0.50 and 0.99 were assumed. For introgression population 1 (**Z** has full column rank), LSQ, BLUP, and RMLV analyses were carried out. For introgression population 2 (**Z** doesn’t have full column rank), BLUP and RMLV analyses were carried out. The sum of correctly detected effects and the sum of false positive effects was recorded for 5000 simulation runs with different random positions of the genes underlying the trait. For the permutation tests *r*=1000 random permutations were used.

### Experimental data sets

We investigated two experimental data sets. The first data set was a rapeseed (*Brassica napus* L.) introgression population consisting of of 350 DH lines. It originates from a cross between the elite line variety Express and the resynthesized line RS239 as donor. The introgression population was genotyped with 484 amplified fragment length polymorphism (AFLP) markers that spanned 1885 cM with an average marker distance of 4 cM. The introgression population covered 100% of the genome of the donor. The lines carried on average 2.8 donor segments, with a mean length of 17 cM. Field trials were conducted at 4 locations in the year 2008/09. Trait data were collected for glucosinolate content (*μ*mol/g) and linolenic acid content (%) measured by using near-infrared spectroscopy. Adjusted entry means were determined with a mixed linear model. The chromosomes in this data set were randomized because the data set is proprietary and the goal of our study is to investigate the analysis methods and not to report QTL for the two traits under consideration.

The second data set was a rye (*Secale cereale* L.) introgression population consisting of 37 introgression lines. It originates from a cross between the elite inbred line L2053-N and the Iranian primitive rye population Altevogt 14160 as donor. The plant height was assessed in two years at five locations with two testers. A detailed description of the experiment is available in earlier publications [5, 12, 21] where the data used in this study is referred to as ‘Library A’. The lines were genotyped with the Rye5K SNP array containing 5,234 markers [22]. The introgression population covered 94% of the genome of the donor. The lines carried on average 4.6 donor segments, with a mean length of 27 cM. This is a public data set, the marker and field data are provided together with the analysis software SelectionTools.

## Results

For introgression population 1 (Figure 1A) and the observation vector shown in Figure 1B, the LSQ, BLUP, and RMLV analyses estimated effects of similar size for all donor segments (Figure 1C). The F-tests for the LSQ analysis as well as the permutation tests for the BLUP and RMLV analyses correctly detected the effect in the center of chromosome 1 as significant and all other donor effects as not significant (Type 1 error rate: 0.01). For introgression population 2, the position of the donor segment underlying the trait was detected correctly by the BLUP and RMLV analyses. However, the BLUP analysis underestimated the effect size considerably. In contrast, the RMLV analysis was able to provide a more precise estimate of the donor segment effect also with the non full-rank design matrix **Z** of introgression population 2.

**Figure 2:**
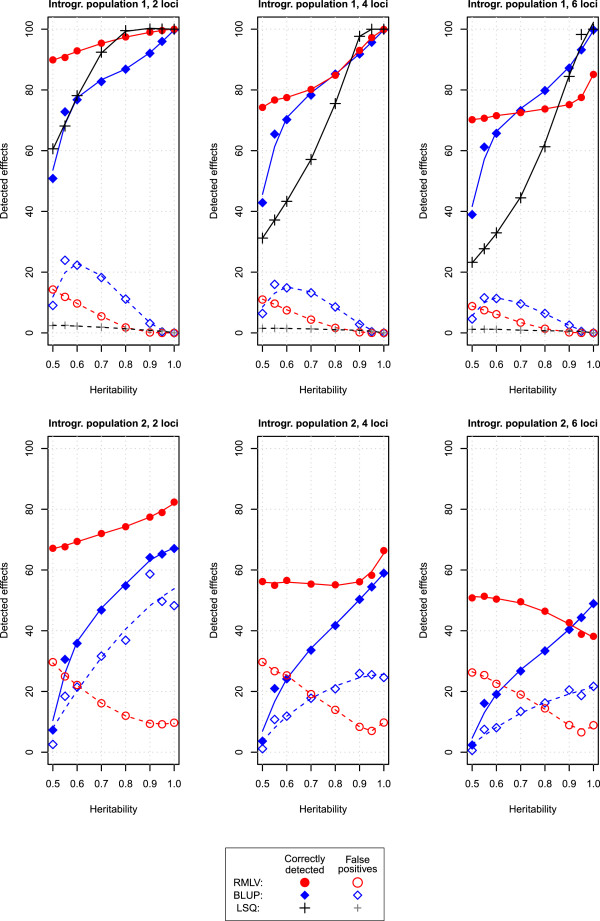
**Correctly detected effects and false positives.** Simulation results for the sum of correctly detected effects (solid lines) and false positives (dashed lines) for the RMLV (red), BLUP (blue), and LSQ (black) analyses of introgression population 1 (top) and for the RMLV and BLUP analyses of introgression population 2 (bottom). Two to six loci were assumed to control the trait under consideration. The heritabilities ranged from 0.50 to 0.99.

In the simulations with the introgression population 1, the LSQ analysis resulted in a false positive rate that was near the nominal type I error rate (Figure 2). The BLUP and RMLV analyses showed greater false positives rates. For heritabilities between 0.6 and 0.8 and four or six loci underlying the trait, the sum of correctly detected effects was considerably greater for the BLUP and RMLV analyses than for the LSQ analysis.

In the simulations with introgression population 2, the RMLV analysis had a greater rate of correctly detected effects than the BLUP analysis for all scenarios with the exception of heritabilities ≥0.9 and 6 loci underlying the trait. For increasing heritabilities, the sum of false positive effects increased for the BLUP analysis while it decreased for the RMLV analysis. The false positive rate of the BLUP analysis was particularly high when only two genes were underlying the trait.

For both introgression populations and all three quantitative genetic scenarios, the RMLV analysis had a considerably greater rate of correctly detected effects than the LSQ or BLUP analysis if the heritability was only 0.5. For introgression population 2 and a heritability of 0.5, the rates of correctly detected effects of the BLUP analysis were below 10%.

**Figure 3:**
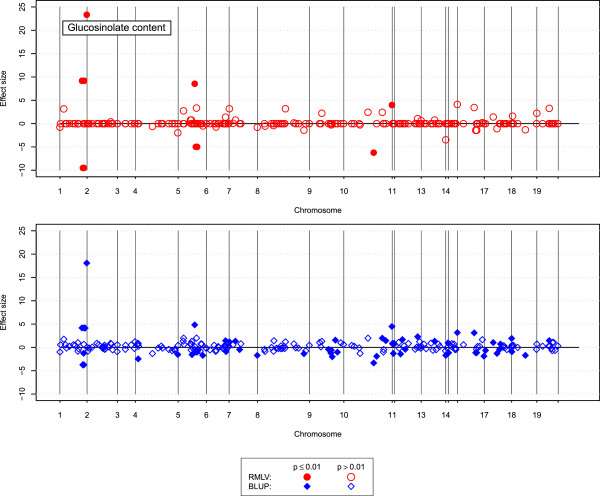
**Donor segment effects for glucosinolate content.** Estimated size of the donor segment effects from BLUP (blue) and RMLV (red) analyses of glucosinolate content (*μ*mol/g) in the rapeseed introgression population plotted along the nineteen chromosomes of rapeseed; filled symbols denote significant effects (*p*≤0.01) and open symbols denote non-significant effects.

**Figure 4:**
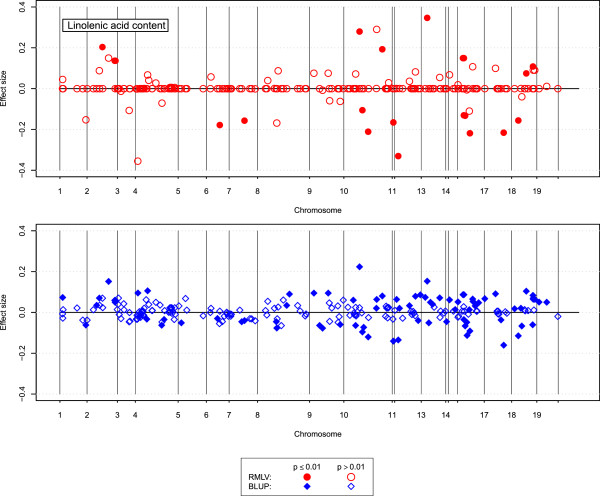
**Donor segment effects for linolenic acid content.** Estimated size of the donor segment effects from BLUP (blue) and RMLV (red) analyses of linolenic acid content (%) in the rapeseed introgression population plotted along the nineteen chromosomes of rapeseed; filled symbols denote significant effects (*p*≤0.01) and open symbols denote non-significant effects.

The RMLV analysis detected that 8 of the 223 donor segments in the rapeseed introgression population were significant (*p*<0.01) for glucosinolate content, the BLUP analysis detected 69 significant segments (Figure 3). For linolenic acid content the RMLV analysis found 25 donor segments, and the BLUP analysis 81 (Figure 4). For both traits the BLUP analysis estimated many small effects, whereas the RMLV analysis estimated a few large effects and many effects near zero.

**Figure 5:**
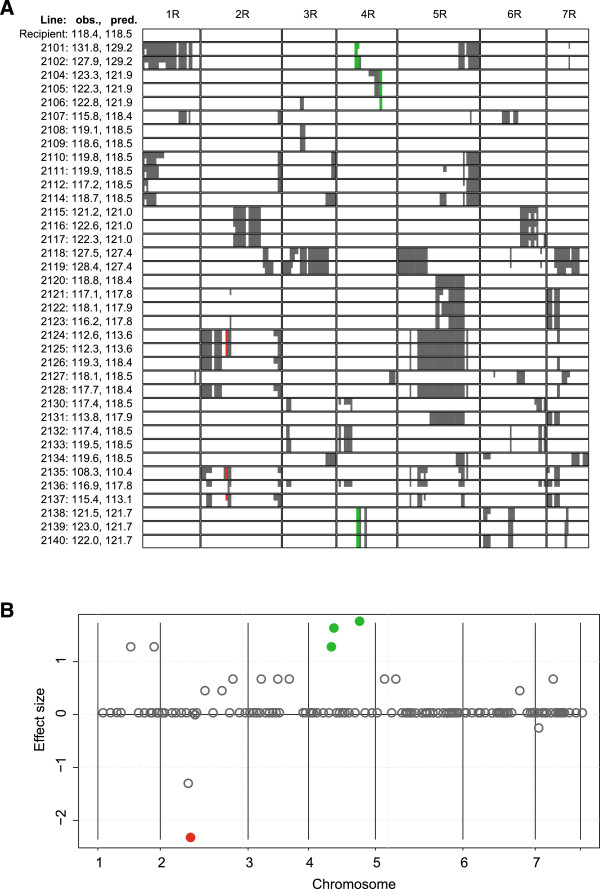
**Donor segment effects for plant height.** **A**: Observed (obs.) and predicted (pred.) test cross values for plant height (cm) of the recipient and the introgression lines 2101 to 2140 of the rye introgression population. In the graphical genotypes white color indicates chromosome segments of the recipient and gray the introgressions from the donor. Green color denotes donor segments that increase plant height and red color segments that decrease plant height. **B**: Estimated size of the donor segment effects from an RMLV analysis plotted along the seven chromosomes of rye; gray circles denote donor segments that are not significant; green color denotes a significant (*p*≤0.05) effect increasing plant height and red a significant (*p*≤0.05) effect decreasing plant height.

In the rye introgression population the RMLV estimation of effects for plant height showed a good model fit, the correlation between observed and predicted values was 0.96 (Figure 5). Three donor segments were detected that significantly increased plant height, and one that significantly reduced plant height. The donor segment that reduced plant height had an additive effect of 2 cm.

## Discussion

### Genome-wide prediction models for the analysis of introgression populations

Combining markers of which the alleles are in complete linkage disequilibrium to donor segments results in a design matrix **Z** with full column rank if (1) the donor segments are non-overlapping, (2) each donor allele occurs exactly in one introgression line, and (3) the donor coverage is 100%. (All three conditions are fulfilled by introgression population 1 in Figure 1.) As a consequence, **Z**^T^**Z** is regular and can be inverted. Hence, in a linear model without intercept the donor segment effects *u*_*i*_ are estimable and can be tested with F-tests for linear contrasts.

For introgression populations that do not fulfill the above conditions (1) to (3), the number of donor segment effects (columns of **Z**) can be greater than the number of lines in the introgression population (rows of **Z**). Because the row rank is smaller or equal to the number of rows, those matrices do not have full column rank, resulting in singular **Z**^T^**Z** matrices. While for such situations the genetic effects *u*_*i*_ are not estimable with ordinary least squares, ridge regression can be employed. Both, the BLUP and the RMLV analyses can be regarded as ridge regression models, BLUP with an equal shrinkage factor for all markers, and RMLV with shrinkage factors, that differ depending on the marker.

Collinearity of the columns of **Z** may occur if conditions (1) to (3) are not fulfilled, and collinearity of the rows of **Z** may occur if strongly related sister lines are among the lines of the introgression population. Such collinearity can increase the false positive rate above the nominal type 1 error rate used for construction of the permutation test. The strength of this departure depends on the strength of the collinearity of the row and column vectors of the **Z**. In conclusion, it can not be expected that the permutation test adheres to its nominal type I error rate, if collinearity is present in **Z**. However, even if the permutation tests are only approximate, they provide a means of analyzing introgression populations that depart from conditions (1) to (3), as do most of the introgression populations that were constructed so far in crops [5, 6, 10, 15, 23, 24].

Typically the vector of phenotypic values **y** in genome-wide prediction models consists of phenotypic means or of adjusted entry means from incomplete block designs. Therefore the residual variance used for the significance tests of the donor segments is only that which is unexplained by the genetic composition, not the full residual variance due to the experimental error of the field trial. This means that the pure experimental error of the plot values is ignored, and the residual variance used in the tests is underestimated. An alternative approach is to adjust the plot values for the effects of the factors that are determined by the experimental design, such as replication, year, or location. Using such adjusted plot values in the genome-wide prediction model results in a more precise estimate of the residual variance. This procedure makes it possible to include the trial design in the analysis, even if the statistical model for genome-wide prediction does not allow to include directly factors for the field design. We applied this approach for our rye data set.

### Power of detecting favorable donor segments and false positive rate

The LSQ analysis adhered in our simulations with introgression library 1 to the nominal type I error rate. However, this was accompanied with a lower power of detecting significant donor segments than the BLUP and RMLV analyses for heritabilities between 0.6 and 0.8 and four or six genes controlling the trait. Hence, with full rank design matrices, the LSQ analysis seems the most suitable method when it can be assumed that the trait is controlled by one or two major genes and the heritabilities are 0.8 or greater. For situations with low heritabilities and in situations where the trait is assumed to be polygenic, the genome-wide prediction approaches might be advantageous for the detection of donor effects, even for full-rank design matrices. The higher type I error rate, however, requires subsequent verification of the detected donor segment effects.

The BLUP analysis showed a very high false positive rate in the simulations with introgression population 2 when two loci controlled the trait. A possible explanation is that the model underlying the BLUP analysis assumes that each donor segment contributes equally to the genetic variance, i.e., the donor segment variances are homoscedastic. This assumption is severely violated if only two genes control the trait under consideration. As a consequence, large effects are underestimated and small or zero effects are overestimated. This systematic estimation error can be observed for the BLUP analysis of introgression population 2 in Figure 1B. The overestimation of small effects is likely the cause for the high false positive rate in the permutation test of the BLUP analysis with non-polygenic inheritance.

The RMLV analysis showed a considerably greater rate of correctly detected effects than the BLUP analysis for low heritabilities. This suggests that an RMLV analysis is an option to detect donor segment effects, which would otherwise remain undetected. Due to the high false positive rate, subsequently a thorough verification of the detected segments is mandatory.

In general, the focus of introgression populations lies on identifying donor segments that have a considerable effect on the trait under consideration. Hence, the traits to be improved are typically oligogenic and are controlled by few major genes. Our simulations have shown that for few genes an RMLV analysis is superior to a BLUP analysis. This is in accordance with the theoretical expectations, because the BLUP approach employs homoscedastic genetic variances at all markers, which can be assumed for highly polygenic traits, but not for oligogenic traits. We conclude that for most applications of introgression populations, where few genes are assumed to control the trait, a BLUP analysis is expected to be inferior to models with heteroscedastic marker variances, such as an RMLV analysis. It remains open to further research how well other heteroscedastic approaches for genome-wide prediction, such as Bayesian methods [17] or the HEM method [25] perform when applied to introgression populations.

A main difficulty of applying genome-wide prediction methods to introgression populations is the rather high false positive rate. It depends on the degree to which the assumptions underlying the statistical models are violated and can not be corrected by adjusting p-values for multiple testing. We therefore conclude that genome-wide prediction methods have the potential to detect favorable alleles, but a validation of the effects in subsequently conducted well-designed trials with a reduced set of lines is mandatory.

### Application to experimental data sets

We applied the BLUP and RMLV analyses to two experimental data sets to derive guidelines for the application of genome-wide prediction methods to introgression populations. In the analysis of the rapeseed introgression population a major gene for glucosinolate content was found, that controls the phenotypic difference between the donor and the recipient (Figure 3). The RMLV analysis estimated an effect size of 23 and the BLUP analysis an effect size of 18. The BLUP analysis detected in addition a large number of significant donor segments with small effects. Many of these were shrunken near zero in the RMLV analysis. The results presented in Figure 1C suggest that the true effect size might be more closely to the RMLV estimate than to the BLUP estimate, because the differences between donor and recipient can mainly be attributed to a single major gene.

For linolenic acid content the BLUP analysis detected considerably more significant donor segments with small effects than the RMLV analysis (Figure 3). Linolenic acid content showed an oligogenic, but not a highly polygenic inheritance in QTL studies [26]. Therefore it can be expected that also here the results of the RMLV analysis are closer to reality than the results of the BLUP analysis.

Plant height in rye showed a polygenic inheritance, but large parts of the genetic variance are controlled by major genes [27, 28]. Therefore, we employed an RMLV analysis for the rye introgression population. The graphical genotypes of the rye introgression lines (Figure 5) indicate that in this data set the rows of the design matrix **Z** show a strong collinearity, because obviously sister lines are included in the introgression population. This might severely violate the assumptions underlying the permutation test. Nevertheless, the RMLV analysis was able to detect a donor segment on chromosome 2 as responsible for the considerably shorter plant height of the lines 2124, 2125, and 2135.

A shorter plant height is a key agronomic property that distinguishes modern rye lines from older breeding material. The exotic donor had a considerably greater plant height than the elite recipient [12, 13, 27]. Hence, the donor segment that reduced plant height found by the RMLV analysis may serve as a proof of concept that favorable alleles can be found in exotic donors, even if the exotic donor itself is inferior to the recipient for a certain trait.

## Conclusions

We conclude that genome-wide prediction methods can be employed to detect favorable donor segments in introgression populations. In particular they can, in contrast to the typically employed Dunnett test [14], identify favorable donor segments when introgression lines carry more than one donor segment and when the segments present in different introgression lines are overlapping. In contrast to fixed linear models, genome-wide prediction methods can also be applied to over-parametrized data sets with non full-rank design matrices.
